# The Role of Felt or Enacted Criticism in Parents’ Decision Making in Differing Contexts and Communities: Toward a Formal Grounded Theory

**DOI:** 10.1177/1074840718783488

**Published:** 2018-06-27

**Authors:** Sarah J. Neill, Imelda Coyne

**Affiliations:** 1 University of Northampton, UK; 2 The University of Dublin, Ireland

**Keywords:** grounded theory, childhood illness, doctor-patient communication, nurse-patient communication, criticism

## Abstract

Felt or enacted criticism was identified as a significant influence on White British parents’ decision making during acute childhood illness in a substantive grounded theory “Containing acute childhood illness within family life.” These parents sought to avoid further criticism, sometimes leading to delayed consultation. Using Glaserian grounded theory principles, we conducted a secondary analysis of data from three studies, to establish the transferability and modifiability of the original theory to other settings and communities in Ireland and England. Felt or enacted criticism was found to operate across the childhood age range, social groups, and settings. Parent’s strategies to avoid criticism reduced contacts with health professionals, access to support and, more worryingly, communication about their child’s health. These findings demonstrate the wider applicability, or “work” in Glaser’s terms, of the concept in the English speaking Western world. Findings indicate the need for nurses to identify and mitigate sources of criticism.

Felt or enacted criticism (FEC) has been identified as having a significant influence on parents’ decision making when seeking help for an acutely sick child at home in the White British population (Neill, Cowley, & Williams, 2013). Parents experience FEC from those in authority, such as, health care professionals (HCPs) when parents breach informal social rules (ISRs). Although HCPs may not intend to criticize, both forms of criticism have been identified in parents’ recall of consultations with HCPs. More empathic communication styles have recently been found to reduce the stress experienced by parents during consultations with a pediatrician (Gemmiti et al., 2017) and may even may result in changes in brain structure and functioning (Wright, 2015). Getting the approach right is important because high levels of stress can have a negative impact on the ability to retain information (Payne et al., 2006). Consultations with health professionals are a frequent event as children constitute a high proportion of the workload in primary care in the United Kingdom, second only to the elderly (Hobbs et al., 2016), and emergency care attendance and hospital admissions are increasing (Keeble & Kossarova, 2017; Neill, Roland, Thompson, Tavaré, & Lakhanpaul, 2018).

The concept of FEC was identified as a key causal category in the substantive grounded theory (SGT) “Containing acute childhood illness within family life” (Neill, 2010). This SGT was identified in a grounded theory study of family management of acute childhood illness at home, involving 29 interviews with 15 White British families of children 0 to 9 years of age in the United Kingdom (Neill, 2008). In this article, the latter is referred to as the original study. FEC was such a key component of the SGT that it was reported separately in Neill et al. (2013) and is referred to herein as the original theory.

The extent to which FEC affects parents’ decision making in different settings and communities is unknown. Therefore, the aim of this article is to explore the transferability of the FEC concept to other settings and communities, such as children’s hospitals in Ireland and other community groups—South Asian and Traveling families, in the United Kingdom.

We used formal grounded theory (FGT) methodology to conduct a secondary analysis of data from three studies. In grounded theory terms, these are the first steps toward a generalizable FGT (Glaser, 2007a).

## Felt or Enacted Criticism (FEC)

Felt criticism is criticism that is perceived and communicated in health care encounters through HCPs attitudes; enacted criticism is direct verbal criticism (Neill et al., 2013). In the original study, parents learnt ISRs from experiences of FEC. Parents described these experiences as being made to feel silly or stupid (Neill, 2008). ISRs are rules of conduct for society, including ceremonial rules and rules of relationships (Denzin, 1970). Criticism was experienced when parents were deemed to have breached those ISRs by, for example, consulting for minor childhood illness which was felt not to require medical attention by the doctor or nurse consulted. Goffman (1972a), in his writings about interactions in social life, provided an explanation for this type of learning; he wrote that individuals become aware of social rules only when they have transgressed and she or he fails to perform as expected and feels shame or guilt. Shame and guilt are unpleasant and therefore parents wish to avoid it in future. It also leads to a fear of such criticism, experienced as a hidden anxiety around any decisions to ask others for advice; particularly those in positions of authority such as nurses and doctors. Such anxiety can lead to delayed consultation and increased morbidity for the child (Neill et al., 2013). The social order or social hierarchy was found to be an antecedent of FEC and, consequently, unequal social power is a condition for FEC. ISRs are ambiguous in modern life (Williams, 2000) adding to parents’ hidden anxiety as they can never assume that ISRs will be similar in any given health care encounter.

## Method

FGT methodology was used to conduct a secondary analysis of data from three studies, to establish the transferability and modifiability of the original theory to other settings and communities in Ireland and England. Data from studies conducted elsewhere, for other reasons, are a major source of data for FGT (Glaser, 2007a).

There are multiple methods for synthesizing qualitative research findings, usually focused on synthesis of published research (e.g., qualitative meta-synthesis, Sandelowski & Barroso, 2007) rather than primary data. The product of these syntheses is often descriptive detailed accounts or thick description (Kearney, 2001). FGTs are generalizable at a conceptual rather than a descriptive level as they are independent of people, time, and place (Glaser, 2007a). A SGT “may have important general implications and relevance and become almost automatically a spring board or stepping stone to the development of a grounded formal theory” (Glaser & Strauss, 1967, p. 79). FEC is the spring board used here for the development of a more FGT as this concept was identified in original interview data from three studies which were available for analysis.

**Figure 1.**
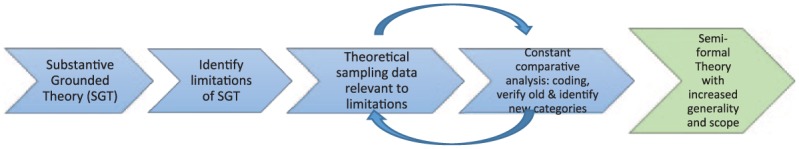
First steps in the development of formal grounded theory.

The method for the development of a FGT employs the same components as SGT: theoretical sampling, theoretical sensitivity, and constant comparison. The one main difference is that theoretical saturation is no longer the goal, as theories can continue to be modified as new substantive theories develop over time. The key steps in the process are outlined in Figure 1.

### Theoretical Sampling

Theoretical sampling in SGT has been defined as “the process of data collection whereby the researcher simultaneously collects, codes and analyses the data in order to decide what data to collect next” (Coyne, 1997) within a substantive site/population. In FGT, Glaser (2007b) explained that sampling is much broader, in other substantive sites and populations, both within and outside the substantive area. Glaser goes on to list a range of different sampling options including the use of existing studies.

Glaser (2007) quoted Strauss as saying that “filling in of what has been left out of the extant theory is a useful first step in extending scope” (Strauss, 1994, p. 371). This first step has been described as improving the “generality and scope” of the theory through collecting data from different sources, which address gaps or limitations in the original substantive theory (Gibson & Hartman, 2014, p. 225). The original theory (FEC) was limited to families with children under 9 years of age in the White British population experiencing acute childhood illness in the home. Therefore, we sought to increase the generality and scope of the substantive FEC theory by sampling other populations: different ethnic groups, children above the age of 9 years, in the home or in hospital, with different types of childhood illness.

According to Kearney (2007), contemporary formal theorists appear to seek models that stay close to the ground (substantive areas) and close to the grounding (in original data). The results, increasingly visible today, are a plethora of substantive semiformal theories closely wrapped in supporting data trails.

Such theory could also be described as “clinically relevant formal theory” presented with data extracts demonstrating the origin of each concept within the theory. One such example is Wuest’s (2001) development of her substantive theory on female caring to generate a midrange theory “applicable to diverse women’s caring in a wide range of health, illness, and developmental situations.” The aim of the work presented here is to extend the scope of the original theory to include a wider range of children, families, and settings and explore how and whether the theory works, fits, and is modified in these differing contexts and communities.

### Studies Included in the Analysis

Three studies were identified which focused on parents’ encounters with HCPs. In all three studies, informed consent was obtained from participants and this consent included the later use of anonymized data for further research and in publications.

#### Choose Well Insight Project

The Choose Well Insight Project, a social marketing project commissioned by Nene Commissioning (National Health Service [NHS] commissioning group now reconfigured as a Clinical Commissioning Group [CCG]), aimed to identify parents’ awareness of, reported pattern and rationale for using, health services for a sick child in an East Midlands town in the United Kingdom (Spencer & Neill, 2013). Data from the qualitative first phase of this project is included in this analysis. Twenty-three mothers with children below 5 years of age from a range of ethnic groups took part in three focus groups in early 2013 (see Table 1). Ethical approval for the project was granted by East Midlands—Nottingham 1 NHS Research Ethics Committee (Ref 13/EM/0016) and governance approval was secured from the local NHS Trust’s Research and Development committee.

**Table 1.** Focus Group Participants.

Focus group	Number of participants	Ethnicity
1	8 mothers	7 × White British 1 × Mixed White and Black
2	9 mothers	3 × Bangladeshi 2 × Polish 4 × White British
3	6 mothers	6 × White British

#### Acutely Sick Kid Parent Information Project (ASK PIP)

The aim of the ASK PIP (part of the ASK SNIFF program of research^1^) was to explore parents’ and professionals’ use of information resources during decision making in acute childhood illness at home (Jones et al., 2013; Jones et al., 2014; Neill, Jones, Lakhanpaul, Roland, & Thompson, 2014; Neill, Jones, Lakhanpaul, Roland, Thompson, & the ASK SNIFF research team, 2015; Neill, Roland, Jones, Thompson, & Lakhanpaul, on behalf of the study group, 2015). Focus groups and interviews were conducted in 2012 with 27 parents in the East Midlands, United Kingdom, from South Asian, and Gypsy/Traveling communities, a Children’s Center and a private sector Day Nursery (see Table 2). Data from HCPs is not included in the analysis here. Approval for the study was obtained from the East Midlands—Nottingham 2 NHS Research Ethics Committee (REC reference 12/EM/0076), the research and development offices of each local Trust and the managers of the day nursery and community centers.

**Table 2.** Ethnic Composition of Focus Groups/Interviews With Parents.

	Traveling community	South Asian community	White British community
Focus groups	5 mothers	South Asian community center: 9 parents (3 fathers and 6 mothers) SureStart Children’s Center: 2 mothers (in a mixed focus group with 1 White British mother)	Day Nursery: 2 focus groups: 2 mothers and 5 mothers SureStart Children’s Center: 1 mother (in a mixed focus group with 2 South Asian mothers)
Interviews	1 mother		Day Nursery: 1 mother Mother’s home: 1 mother
Number of participants	6	11	10

#### Family Centered Care Project

The aim of the Family Centered Care Project was to explore parents, children, and nurses’ experiences of family-centered care and how they negotiate roles and relationships within an inpatient setting in Ireland (Coyne, 2015). Ethical approval was obtained from the three hospital and university ethics committees. Individual audio-recorded in-depth interviews with 18 parents, from two general medical and surgical wards in two children’s hospitals and one surgical children’s ward in a district general hospital in Ireland, is included in the analysis (see Table 3). Their children’s ages spanned from 7 to 16 years with a mean age of 12 years.

**Table 3.** Ethnic Composition of Interviews.

Sites	Number of participants	Ethnicity
Site 1	6 mothers	6 × White Irish Aged 35-43 years
Site 2	5 mothers 1 father	6 × White Irish Aged 33-45 years
Site 3	6 mothers	6 × White Irish Aged 37-46 years

All three projects included conversations with parents concerning their interactions with HCPs and the impact on their help seeking behaviors. Analysis of this data provided insights into the underlying social structures, contributing to the development of a semiformal theory with greater generality than that from any one of the original projects.

### Data Analysis

**Figure 2.**
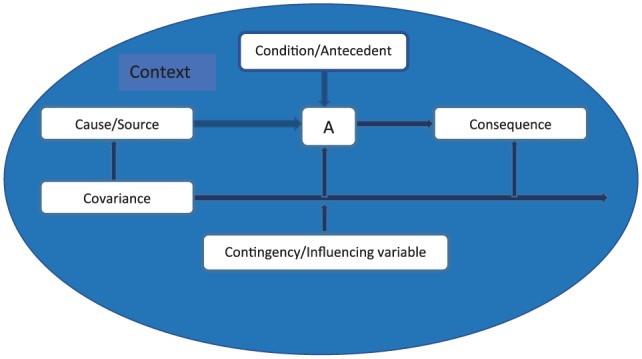
Glaser’s (1978) 6Cs coding family.

The focus of analytic activity during the development of FGT is on comparison of concepts, categories, subcategories and their properties, rather than on descriptive comparison of data. Data from the three studies above was coded to existing categories within the original FEC theory first, remaining theoretically sensitive to the emergence of new concepts, categories, and relationships during the process. This process facilitated the development of additional subcategories within the original theory. As with the development of SGT, activity cycled backward and forward between theoretical sampling of data available and constant comparative analysis as concepts are compared, questions raised, and data sought to answer questions and clarify categories (see Figure 1). Glaser’s (1978) 6Cs coding category (see Figure 2 below) was used to structure the analysis; this includes conditions (or antecedents), causes (including sources), consequences, context, contingencies (or variables), and covariances (variables which are connected, changing together, without a causal connection). The 6Cs framework facilitated the development of a more detailed coding frame than that developed for the original FEC theory. See Table 4 where “^a^” denotes new subcategories.

**Table 4.** Felt or Enacted Criticism Categories and Subcategories.

6Cs Coding category	Categories and subcategories
Conditions/antecedents	Social expectations/informal social rules • Conforming/doing the right thing
	Social order/hierarchy • Influences on social distance^a^ • New migrant status^a^
Causes/sources	Unequal power • Challenging the social order • Professional’s attitudes • Intimidation^a^
	Discrediting attributes • Identified^a^ • Hidden^a^
	Labeling
	Discrimination^a^
	Social actors/people • Family members • Child carers • Nursery and School staff • Health care professionals (doctors, nurses, health visitors, midwives)
Consequences	Hidden anxiety/fear of criticism • Taking control • Delayed consultation • Reduced communication^a^ • Information seeking • Legitimating help seeking
	Loss of control • Reduced self-efficacy • Increased need for help • Reduced self esteem
Context	Community • Home • Primary care
	Hospital ward^a^
Contingencies/influencing variables	Relationship length • Trust^a^
	Parental knowledge and confidence
	Severity of the child’s illness
	Urgency of child’s physical needs^a^

a New categories or subcategories identified during this secondary analysis.

### Theoretical Sensitivity

Researchers engaged in the development of FGT each have a unique standpoint or theoretical sensitivity developed from their unique history, culture, methodology, sociological knowledge, and so on, just as they do when developing SGT. Kearney (2001) suggests that, when studies completed by other authors are included in a FGT, the paradigmatic perspective of these authors must be included in the FGT. In the work toward a FGT reported here, the researcher involved in two of the three studies included was the originator of the starting point SGT (S.N.). The third study was led by the second author (I.C.). Both authors are academic children’s nurses sharing some professional perspectives and expertise in grounded theory methodology. In addition, paradigmatic perspectives were shared during the analytic process through discussion of each concept.

## Results

Experiences of criticism were identified in each of the projects included in this analysis. Most often, these were reported to be felt criticism rather than overt enacted criticism, as previously identified in the original substantive grounded theory (Neill, 2008):

so when you’re talking to somebody you don’t (want to) feel as if, “Oh, I’m being really stupid here,” you know, because I always feel like that, I always feel like, “Oh, am I being really silly?” you know, because I don’t want to waste peoples’ time and . . . (White British mother in day nursery)

The key causal category remains “felt or enacted criticism.” Table 4 sets out the categories and subcategories identified from our analysis of the FEC concept, its antecedents, sources, consequences, contexts, and contingencies/influencing variables. No covariances (variables which change with the key causal category) were identified.

### Antecedents or Conditions

These are the conditions which were identified as existing when FEC was reported. Two broad categories of antecedent were identified: social expectations, often referred to as ISRs, and social hierarchy, represented in the degrees of perceived social distance between parents and HCPs. Social distance is the degree to which people feel they are socially inferior or superior to another person, usually someone in authority.

#### Social expectations

Social expectations or ISRs, those everyday unwritten rules of social life create the rule frame for expectations of parents when their children are sick. In primary care, these rules govern when parents are expected to seek help for a sick child, while in hospital they are represented in the social rules for parent’s behavior when they are present on the ward with their child, including expectations that they will be present whenever possible. Parents want to do the right thing for their child and in the eyes of others and therefore attempt to conform to these ISRs. One Irish mother in the hospital setting explained that “no matter how much more I might want to do for D I couldn’t do it unless the nurses said I could.”

#### Social hierarchy

The existence of a social hierarchy presented social conditions within which HCPs communicated with parents in a manner which was perceived as criticism by parents, sometimes reported to be open, enacted criticism, but more often nonverbal communication which was experienced as felt criticism. The greater the social distance, the more likely parents were to report FEC and the greater their fear of future such criticism. This made it more difficult to raise concerns with those in positions of greater power. This unequal power was identified as a cause of FEC and it is discussed further later in this article.

Some parents experienced greater social distance between themselves and their HCPs; these were groups who felt HCPs had labeled them as less competent parents such as Gypsy/Traveling families or mothers who felt they had been labeled as neurotic or overprotective, and those whose social circumstances had reduced their social capital such as new migrants. This latter group may have been qualified professionals in their country of origin but were not able to work in that capacity in the United Kingdom. The resulting disempowerment was reported to also affect their ability to advocate for their child and communicate with health services.

### Causes or Sources

The source of FEC reported by parents was, as might be expected given the focus of the three studies, HCPs: doctors, nurses, health visitors, and midwives. In the original grounded theory, study parents also reported criticism from family members, nursery, and school staff. These people should be viewed as social actors whose behavior is shaped by the social order and its ISRs, as are parents, albeit with greater power in each encounter than the child’s parents.

**Figure 3.**
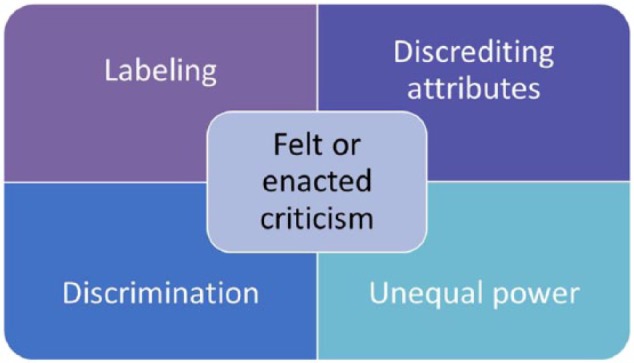
Key sources of felt or enacted criticism.

Four key sources were identified: people acting in the context of unequal power, labeling, discrediting attributes, and discrimination (feared rather than reported)—see Figure 3. As in the original SGT, these four sources reflect the key components of stigma identified by Link and Phelan (2001), although parents do not report stigma.

#### Unequal power

Unequal distribution of power between parents and HCPs was, as might be expected, identified as both a consequence of the social hierarchy and a cause of FEC. HCPs higher social status was also reflected in parent’s report of their attitudes toward parents, dismissing their concerns about their child or failing to explain their decision making. In some more extreme examples, parents reported situations where they felt intimidated by HCPs:

You feel intimidated by doctors more because the doctors, you think that they know everything but you know that what they’re saying is not what you’ve got, but you feel like you can’t argue the point with them. (South Asian mother in community center)

You might be a wee bit put upon to speak up because when they do the big round, it’s half 7 in the morning, you’re kind of lights on all of a sudden and it’s awfully early . . . It’d be very disorientating in this light all of a sudden, about 20 people at the bottom of your bed or 10 people and you can barely get to speak. (Irish mother in hospital)

Parents are aware of the social status of nursing and medical staff and report worrying about “undermining their (HCPs) professional status” (Irish mother in hospital). Where parents attempted to challenge the social order by raising concerns about the quality of care received by their child one Irish father told “I would be removed physically” (In hospital) or in the original study in primary care (Neill, 2008) parent’s request for specific treatment was rejected.

The unequal power in the relationships between parents and HCPs appears to enable HCPs to act as moral agents judging parent’s behavior when their children are sick—measuring that behavior against the HCPs perception of ISRs. Parents’ perception of unequal power is greatest when the *social distance* is greatest, for example, between doctors and parents, and less when the interaction is between a nurse and a parent.

You get so many doctors, especially males, that are, I think they’re rude and they make you feel like you’re a timewaster when actually you know there’s something wrong with your kid and you’re not wasting their time and you know, they could send you home and anything could happen. (White British mother in community center)

The greater the social distance the more difficult it seems to be for parents to communicate with HCPs. The *setting* within which the encounter occurs also seems to act as an aggravating factor—in the hospital setting the inequality in power distribution is increased by parent’s *loss of control* in the unfamiliar environment and as a consequence of not being able to restore their child to health themselves. Parents report feeling powerless and socially isolated. “It was a very overwhelming feeling coming into hospital with your child . . . I am totally dependent” (Irish mother in hospital).

Parent’s powerlessness or lack of agency is particularly visible in the hospital setting in the poor facilities provided for parents (“I didn’t even know there were showers available for parents, even though I was sleeping down here.” Irish mother in hospital), the lack of information from staff about their child (“I wasn’t being told stuff about my son, I overheard conversations outside the door to other doctors . . .” White British mother about hospital experience; “They don’t tell me enough, ever and that frustrates me . . .” Irish mother in hospital), about ISRs for parents in the ward setting (“You don’t know how far you can go, you don’t know what’s expected from you or not . . .” Irish mother in hospital) and the way in which HCPs time is prioritized over that of the child and family:

You are always told “the doctors will be around,” but when will they be around . . . because you are always sitting here waiting for them . . . you are never told when . . . and I understand that’s hard but, but it’s difficult for us as we are sitting waiting afraid to go for food or . . . (Irish mother in hospital).

#### Discrediting attributes

Parents only become aware of ISRs when they have transgressed (Goffman, 1972a), this transgression becoming a discrediting attribute for that parent, damaging their moral status as a good parent. Transgression is communicated to parents through FEC. In the community, the discrediting attribute may be seeking help for minor illness, giving or not giving their child basic medical care—“He (the paramedic) was like, ‘Well, if you knew he had a fever you should have given Calpol,’” South Asian mother in community center—or consulting too late for serious illness. In the hospital, it may be transgression of hidden ISRs concerning parents’ role in the ward setting. Parents, therefore, wish to avoid being discredited or labeled and will attempt to control their exposure to scrutiny to avoid breaching ISRs and/or to keep any attributes that they feel may be discrediting hidden. Mothers in the Irish hospital setting talked about having to “gently see if this or that is OK” and being “afraid to do anything” or of being “a little bit frightened of doing it in case somebody says you shouldn’t.” The consequences of FEC are discussed in more detail below.

#### Labeling

Some parents reported feeling that they have been labeled (as mentioned above), or may be labeled as a neurotic or over anxious mother, an inadequate young parent, a demanding parent or as belonging to a discredited group such as the Gypsy/Traveling community. “You don’t want to be coming across as a neurotic over-protective mother” (Irish Mother in hospital).

“You don’t want to be *labelled* as a fussy or a demanding parent” (Irish Mother in hospital). “*They all paint us with one brush*, they (doctors) have no time for us, they thinks it’s just travelers being bad” (Gypsy/Traveler mother in community center).

This labeling appears to act as a form of instant discredit, which results in HCPs taking parent’s stories about their child’s illness less seriously.

But some doctors do think that because you younger, they just *fob you off*. (Gypsy/Traveler mother in community center)

You are made to feel like an over sensitive mother . . . oh don’t worry about them sure I get them . . . what kind of a response is that to give a parent . . . (Irish mother in hospital)

#### Discrimination

Discrimination was not reported but feared. Parents appeared to fear that if they do not conform to ISRs, their child’s health care will be affected. In the community, this leads parents to seek advice through sources with least risk of criticism rather than seeking a re-consultation.

We’ve all taken a sick child to the doctor only to be pooh-poohed away, you know, Calpol for the next 2 days and the child will be fine and then you feel silly . . . So I think you get a reluctance that builds up. (White mother in the original SGT)

You actually feel that, you know, the doctor says “oh this is really minor you didn’t really need to come here.” (South Asian father in community center)

In hospital, parents avoid “bothering staff” resulting in reduced communication. As mentioned above parents carefully navigate the hospital setting to avoid breaching any ISRs. This can also lead to fear of discrimination: “You are sometimes *afraid to say* things to nurses in case C’s care would be affected in any way . . .” (Mother in hospital). In all settings, though, parents report that they will speak out if they feel their child’s life is at risk. Although that strategy is not always successful as reported above. Parents’ experiences in both settings were sometimes close to stigmatization as they reported unequal power, labeling, and wanting to avoid displaying discrediting attributes and, a fear of discrimination. As originally suggested (Neill, 2008) FEC appears to be a less severe form of felt and enacted stigma (Scambler, 2004; Scambler & Hopkins, 1986).

### Consequences

Two categories were identified as consequences of experience of FEC: hidden anxiety and loss of control.

#### Hidden anxiety

Parent’s worry about being perceived to have done the wrong thing creates a hidden anxiety or unspoken fear of FEC, similar to the concepts of felt stigma and hidden distress described by Scambler and Hopkins (1986). In the hospital ward setting, Irish parents reported worrying about “overstepping the mark”—“I would be quite cautious about over stepping the boundaries.”

Parents desire to avoid being criticized and therefore avoid being discredited for breaching an ISR leads to the use of a range of strategies to take control to avoid exposure to scrutiny. Strategies identified in the data include delaying consultation, seeking information from a range of sources (see Neill et al., 2015, for detail on parent’s information seeking), legitimating their need for health consultation for their child, using health services with least risk of criticism such as accident and emergency department where they are not known and reducing communication with HCPs by keeping quiet. “. . . Sometimes I have questions but the nurses are busy and I don’t feel I want to ask them or annoy them about it” (Irish mother).

This anxiety is related to the ambiguity of ISRs (“Anxiety happens because people don’t know where they stand.” Irish mother in hospital) which leaves parents uncertain about how they are supposed to behave as a “good parent.”

Parents are using impression management when they use these strategies to avoid criticism and present themselves as a “good parent,” protecting their moral identity by attempting to conform to social expectations. Parents have been found to engage in “facework,” in Goffman’s (1972b) terms, to manage the impressions they create in a range of social settings, from the school gate (Guendouzi, 2005; Neill, 2008) to within health care encounters (Neill, 2008; Smart & Cottrell, 2005; Todd & Jones, 2003).

#### Loss of control

Receiving criticism acts as a deterrent to using that source of help in the future, increasing parent’s feelings of loss of control of their child’s illness, as they feel an avenue of support is no longer available, yet they still feel they need help to manage their child’s illness.

Whereas my doctors, I went in once . . . and she actually told me off for coming in, she was like, “Well I haven’t got time for you,” and I come out just feeling really, and I wanted to ask a couple of questions but I felt I couldn’t ask them. (South Asian mother in community center)

In the original study, young parents talked about how their experiences of criticism also reduced the mother’s self-esteem—making her feel as if she was an inadequate mother (Neill, 2008). In the analysis reported here, the data indicate that criticism and the fear of it can lead to loss of self-efficacy, particularly in the disempowering setting of the hospital ward where they are trying to navigate ISRs:

. . . the nurse came in and asked him if he had had any pain relief all day and she looked at his chart and said “oh my god you poor thing you haven’t had any pain relief all day” . . . and then again I got hit with the guilt again . . . and I thought oh my God am I an awful stupid woman why did I not think about pain relief . . . but I didn’t realize that I could ask them for some pain relief and that they didn’t want anyone in pain . . . you know it’s a balance knowing what you can ask for and what you can’t ask for. . . (Irish mother in hospital)

Not knowing if it was acceptable to ask added to the loss of control this mother felt as a result of being in hospital with her child. It reduced her ability to meet her child’s needs for pain relief although she also said she would have given it had she been at home.

### Context

The example given above shows how the setting within which parents encounter HCPs can have a significant effect. Comparison of data across these three studies suggests that environments where parents have increased loss of control or lack of agency, such as the hospital ward, add to their hidden anxiety or worry about doing the wrong thing. This loss of control also affects their ability to use strategies to reassert control. In the hospital setting, parents are unable to independently seek information about their child’s illness or care, or to access their usual support mechanisms through which they would usually discuss the legitimacy of raising their concerns. The only strategy left is to keep quiet, avoid bothering busy staff, resulting in a reduction in two-way information sharing with HCPs. Although most of the data illustrating these effects is drawn from the hospital setting, there are indications that any factors which reduce parent’s social agency, such as being a new migrant, is likely to have similar effects. Context in this analysis could also be viewed as an influencing variable.

### Contingencies or Influencing Variables

Parent’s knowledge and confidence, relationship length, severity of the child’s illness and the urgency of the child’s physical needs were all identified as contingencies or influencing variables as each of these were found to change parent’s response to fear of, or experiences of, FEC.

Parental knowledge and confidence and relationship length were interrelated in the hospital setting: Parent’s knowledge and confidence was reported to increase as their knowledge of the ward environment increased and their relationships with health professionals developed, enabling parents to raise concerns about their child’s health with less fear of criticism.

I do think that the longer you are here you would become more comfortable with the place . . . you would probably feel more comfortable to get involved. (Irish mother in hospital)

I suppose you get to know the actual staff that are looking after him as in the doctors and . . . the nurses on all of the different wards and they would know me too, so yeah I would say my confidence would certainly have increased . . . and that would certainly have enabled me to stand up and want to know more rather than just listen and take on board what they say. (Irish mother in hospital)

We made it clear now that we need to know what is happening, but it has taken us time to be able to stand up for ourselves and our daughter . . . it has been a struggle, I just feel that we were such meek parents the first time that we came in and we were so nice and just said yes or no to everything and I now feel that you have to be forceful to be listened to and it’s not what you want to be but sometimes to get answers that’s how you have to be and then all of a sudden they are all down here rallying around you . . . you shouldn’t have to be like that, I shouldn’t have to be doing that and you don’t want to turn people against you either, which can easily happen . . . we haven’t so far but believe me there are times . . . (Irish mother in hospital)

As trust developed between parents and HCPs, all actors in the relationship were able to share more information and to trust that information. In primary care, this influence was undermined by parent’s report of the lack of continuity:

It’s just . . . why would you go and seek help from the Health Visitor when you’ve only actually had one visit from her and you really don’t know her that well. (Gypsy/Traveler mother in community center)

. . . There’s no continuity of care. So how would they know whether that was normal or not normal for your child? So that’s one of the reason why I think I don’t always trust them as well, because of the continuity of care. (South Asian mother in community center)

When parents felt that their child’s illness had increased in severity parents reported over-riding their fear of criticism and raising concerns to protect their child:

At first, we just went with whatever the doctors were saying, as you do as a parent, but then we just felt unhappy with her care and started to ask questions and here we are . . . at that stage we were scared, to be honest, because we were imagining all sorts of things could be wrong with her. . . (Irish mother in hospital)

This was not universal though, as there were examples of parents who continued to be unable to advocate for their child in the hospital setting. This suggests that unequal distribution of power may be a more important deterrent to parent’s raising concerns than their worries about their child’s illness. This may explain what appears to be a greater impact of social hierarchy in the hospital setting, where parents are disempowered and do not know the ISRs, compared with the home or primary care.

Interestingly, parents seemed to find it easier to ask for help or to take action to meet their child’s physical needs, than to raise concerns about their child’s illness and treatment:

I feel her (physical) needs were not being met and so I started to tend to them . . . and if they did have a problem I would just say “listen we have been sitting here for ten minutes waiting for a bedpan, this is not good enough and I would prefer to just fetch the bedpan myself if it’s alright.” (Irish father in hospital)

Perhaps it is easier to justify asking for help with physical needs such as toileting than asking questions or raising concerns related to the severity of their child’s illness. Parents know they are not the experts on illness but they are experts on their child. It may also be related to the greater clarity of ISRs around parent’s responsibility for the physical care of their child.

## Discussion

FEC appears to operate across settings, social groups, childhood illnesses, and age ranges. Social hierarchies and ISRs (social expectations) were identified as antecedents for FEC in this more delimited context. The four key causal categories for FEC were identified as unequal power, labeling, discrediting attributes, and (fear of) discrimination. These categories continue to reflect those identified for stigma as in the original SGT. Discrimination was, as before, not reported but feared. This analysis has further identified the greater magnitude of effect where social distance is greater and parent’s loss of control higher. Hidden anxiety was therefore increased for parents in the hospital setting and might be expected to be more pronounced for groups such as new migrants where the social distance between parents and HCPs is magnified. This anxiety does appear to shape parent’s decision making, leading to strategies to avoid the risk of criticism, including delaying consultation, reducing communication with HCPs, seeking legitimation of their need for help and information seeking. All these strategies were used across social groups in the community setting. In the hospital setting, parent’s options were reduced by their physical relocation to the hospital setting which, in itself, reduced their control of events and their self-efficacy, leaving them with only the option to reduce communication—presenting themselves as the compliant quiet conforming parent and, in doing so, protecting their moral identity. Variables which emerged as influencing parent’s response to fear of, or experiences of, FEC included parental knowledge and confidence, relationship length and trust in their HCP, perceived severity of their child’s illness, and, in the hospital setting, the urgency of their child’s physical needs. Gender was also identified as a variable in the original SGT but was not identified in this analysis.

Overall, the theory of FEC, its antecedents and consequences, was found to work across all three studies in the analysis. A more detailed coding frame was developed, which in turn generated a greater depth of understanding of concept, clarifying relationships within and between categories.

### Implications for Practice and Policy

There are important lessons here for nursing practice and policy. Nurses and other health professionals need to be aware that parents are sensitive to criticism in their interactions, whether it is communicated verbally as enacted criticism, through nonverbal communication or through signage which implies that parents have, or may, somehow breach(ed) ISRs. This is particularly important in the hospital setting where parents have minimal control, the power imbalance is greatest between parents and professionals and a trusting therapeutic relationship is essential (Coyne, 2015; Dennis, Baxter, Ploeg, & Blatz, 2017; Shields, 2016). Nurses need to be aware that the first interaction and how they approach the family can set the tone for all future interactions, be it positive or negative. It is clear that perceived criticism can hamper parent’s efforts to cope with the stress of hospitalization and leave them reluctant to ask for help, leading to unmet needs and potential safety issues (Coyne, 2008; Rosenberg et al., 2016). We need to find ways to mitigate and replace the damaging effects of enacted criticism with practices that invite health and healing in family members and families as the unit of care and which involve addressing the emotional, social, and spiritual needs of family members and family units. To avoid further stress and disempowerment of parents, HCPs need to find ways to promote positive communicative interactions between themselves and families (Foster, Whitehead, & Maybee, 2016). There is a considerable body of research indicating the benefits of family nursing conversations (Bell, 2016). The family nursing 15-Minute Family Interview is a useful aid to begin the process of “therapeutic conversations” and a gentle way of inviting families to express their feelings and needs, thereby contributing to mutually respectful relationships (Bell, 2012). Nurses could also use the simple process of “ask-tell-ask” which begins with asking parents what they know and building upon that while checking constantly for mutual understanding of issues discussed.

The findings indicate that nursing policy at national and local level needs to emphasize the competencies necessary for a nonjudgmental empowering approach to parents. Nurses need education and guidance on how to develop and practice relational and communication skills with families (Bell & Wright, 2015). The International Family Nursing Association (IFNA) has recently developed the IFNA Position Statements on Generalist Competencies for Family Nursing Practice (IFNA, 2015) and the IFNA Position Statement on Advanced Practice Competencies for Family Nursing (IFNA, 2017) that highlight the beliefs, knowledge, and skills needed by HCPs. The statements emphasize the importance of collaborative relationships and therapeutic conversations between nurses and families that focus on strengths and acknowledge different realities. The National Academy of Medicine has published a useful guiding framework for thinking about how to achieve culture change for patient and family engaged care (Frampton et al., 2017). They also identify that skilled practice is influenced and enhanced by the culture, infrastructure, and particular skilled practices and tactics, that can be learned, that will address these emotional, social, and spiritual needs of family members and families.

### Limitations

The strengths of this comparative analysis using the grounded theory approach are in its rigor and its ability to extend an existing SGT beyond its original limited setting to other contexts and communities. This process demonstrates the modifiability of the original SGT, enabling the identification of concepts, which persist irrespective of time and place while also further clarifying the categories within the theory of FEC. The availability of the original data from the three studies has facilitated the ability to substantiate the resulting analysis with exemplars from primary data, rather than the more abstract analysis sometimes used to develop FGT (Gibson & Hartman, 2014; Glaser & Strauss, 1967). Glaser (2007b) warns the formal grounded theorist of the pitfall of “falling into description comparison” instead of remaining focused on the core category. We have endeavored to do this while also providing sufficient description to support our theoretical developments.

The collaboration of the lead researchers from the three studies has also enabled sharing of their paradigmatic perspectives and rich discussions around the development of the theory. The result is, therefore, a theory which remains grounded in original data and close to the “ground” in Kearney’s (2007) terms—the substantive area of parent’s interactions with HCPs during childhood illness.

The main limitation of this work is in the reliance on reanalyzing extant data for “fit” (in Glaser’s terms) to the original theory. Although it is reassuring that “fit” was identified across these studies and the original theory further developed, it is also possible that in selecting these studies we did not identify work with contrasting findings. Glaser (2007b) recognized that “theoretical sampling (in FGT) depends on availability of data” (p. 79) which may be within existing studies. Further data may provide opportunities for additional theory modification.

A range of different strategies for the development of FGT have been identified (Gibson & Hartman, 2014; Glaser, 2007b; Kearney, 2007), creating a degree of ambiguity. Yet this ambiguity also allows for creativity in the use of available data so long as it is subjected to the constant comparative conceptual analytical process at the heart of the development of FGT.

The work remains limited to an English speaking European population and data drawn from small samples, dominated by mothers, in qualitative research. Consequently, the father’s voice in the analysis was limited, which may explain why gender was not identified as a variable. However, the diversity within the samples included do demonstrate the “fit” of the theory across a range of communities and clinical settings, with differences emerging only in the degree of effect. The result is a substantive semiformal theory.

## Conclusion

FEC was found to operate in all three studies demonstrating its applicability, or “work” in Glaser’s terms, across the childhood age range, social groups, and contexts in the English speaking Western world. The concept was often expressed in the context of perceived social hierarchy within which nurses and doctors continue to be seen as socially superior to parents. Where this social distance was greater, and/or parent’s loss of control higher, FEC appeared to have more impact, leading to increased hidden anxiety for parents in the hospital setting. Parent’s use of strategies to avoid criticism varied more between settings than between social groups, illustrating the modifiability of the theory.

Experiences of FEC, and the hidden anxiety created, led parents to reduce contacts with HCPs, reducing access to support and advice and, more worryingly, reducing communication about their child’s health. The consequences of the latter are that important information about their child’s illness is not shared with HCPs, who are then not able to use this information to inform their diagnosis and treatment decisions. Nurses, doctors and other HCPs are also likely to be influenced by social expectations in enacting their roles. No evidence was identified which suggests that HCPs intend to criticize parents, indicating that they are unaware of their impact on parents across settings. It is clear that a more empowering approach is needed in HCP’s encounters with parents of sick children, which recognizes the potentially disempowering effect of social hierarchies and ambiguous ISRs. Health professional education should include education concerning how the impact of negative communication styles and how to engage in positive empathic conversations with parents such as the 15-Minute Family Interview (Bell, 2012; Wright & Leahey, 2013) or Fisher, Broome, Friesth, Magee, and Frankel’s (2014) brief intervention for newly licensed/qualified nurses. All health professionals should receive this education, not just novice nurses, as the experience of FEC is greater where there is increased social distance.

### Directions for Future Research

Further research is needed to determine the specific HCP behaviors perceived to be critical by parents so that evidence-based educational interventions can be developed for HCPs.

A range of tools have, and are, being developed to help parents to know when to seek help for their children when they are sick, such as the work of the ASK SNIFF team and the development of digital “apps” by charities such as Meningitis Now. These tools are an attempt to “fix” the problem of parents’ lack of knowledge and/or help seeking behaviors not deemed appropriate by HCPs, without consideration of the reasons for parent’s actions. Instead, we contend that the focus of future research should be on HCPs behaviors to remove at source FEC. It is these behaviors, which result in parents avoiding, and sometimes delaying, consultation with HCPs.

The original SGT has been extended in scope and generality, demonstrating its transferability beyond the original context and community. The detailed coding frame developed makes the phenomenon identifiable and researchable in any setting or community. This coding frame can now be used as a tool to establish the generalizability of FEC to diverse groups beyond family nursing, even beyond health care, to any context where social expectations and social distance exist between interacting parties—a significant step toward a FGT of FEC.

## Notes

1. ASK SNIFF Acutely Sick Kid Safety Netting Interventions for Families, www.asksniff.org.uk
